# The C3d-fused foot-and-mouth disease vaccine platform overcomes maternally-derived antibody interference by inducing a potent adaptive immunity

**DOI:** 10.1038/s41541-022-00496-8

**Published:** 2022-06-28

**Authors:** Min Ja Lee, Hyun Mi Kim, Sehee Shin, Hyundong Jo, So Hui Park, Su-Mi Kim, Jong-Hyeon Park

**Affiliations:** Animal and Plant Quarantine Agency, 177 Hyeoksin 8-ro, Gimcheon-si, Gyeongsangbuk-do 39660 Republic of Korea

**Keywords:** Viral infection, Inactivated vaccines, Conjugate vaccines

## Abstract

Vaccination prevents and controls foot-and-mouth disease (FMD). However, the current FMD vaccine remains disadvantageous since it cannot overcome maternally-derived antibody (MDA) interference in weeks-old animals, which suppress active immunity via vaccination. To address this, we developed the immune-enhancing O PA2-C3d and A22-C3d FMD vaccine strains that can stimulate receptors on the surface of B cells by inserting C3d (a B cell epitope) into the VP1 region of O PA2 (FMDV type O) and A22 (FMDV type A). We purified inactivated viral antigens from these vaccine strains and evaluated their immunogenicity and host defense against FMDV infection in mice. We also verified its efficacy in inducing an adaptive immune response and overcome MDA interference in MDA-positive (MDA(+), FMD-seropositive) and -negative (MDA(−), FMD-seronegative) pigs. These results suggest a key strategy for establishing novel FMD vaccine platform to overcome MDA interference and induce a robust adaptive immune response.

## Introduction

Foot-and-mouth disease (FMD), an acute infectious disease in cloven-hooved animals, especially pigs and cattle, causes significant economic loss to the livestock industry as it rapidly spreads, thereby causing high mortality in young individuals and reducing productivity^1,2^. The current commercial FMD vaccine requires periodic and repeated vaccination in both cattle and pigs. Following vaccination, the maternally-derived antibodies (MDA) are transferred to the offspring through the placenta or ingestion of colostrum to form passive immunity. Upon initial infection with the FMD virus (FMDV), the MDA have a short-term protective effect in calves and piglets. Early vaccination of an FMD vaccine in young-week-old animals causes interference via passive immunity by inhibiting antigen-specific antibody production in plasma cells and memory B cells, resulting in immunological tolerance, which reduces the efficacy of the vaccine and inhibits the formation of active immunity^3^. Therefore, the current FMD vaccination program in Korea recommends that calves and piglets be vaccinated 2–3 months after birth, when the MDA levels decrease. Since the level, titer, and half-life of MDA vary between individuals, it is difficult to determine the appropriate timing for FMD vaccination in practice. Moreover, the commercially available FMD vaccine cannot overcome the interference by MDA.

Various studies have reported the relationship between MDA interference and reduced efficacy of FMD vaccines^4–6^, and the optimal timing for vaccination in young animals^7,8^. However, few studies have suggested strategies for inducing a strong immune response by effectively overcoming MDA. Vaccines are also being developed against other viruses, such as NDV^9,10^, AIV^11^, PRRSV^12^, PCV-2^13^, IAV^12^, and CSFV^14^, to overcome MDA interference in birds and pigs. However, few systematic studies with an immunological approach have been conducted on the development of a vaccine composition that can simultaneously induce a strong cellular and humoral immune response while evading MDA interference.

There are three main pathways for the activation of B cells: 1) the T cell-dependent pathway, 2) the T cell-independent pathway (type I), and 3) the T cell-independent pathway (type II). In the T cell-dependent pathway, B cells are activated through the TCR/MHC complex and the CD40L (CD154)/CD40 pathway, among others. In the rare T cell-independent pathway type I, a pathogen-associated molecular pattern (PAMP) stimulates pattern-recognition receptors (PRRs) to directly activate B cells. In the T cell-independent pathway type II, B cell receptors (such as CD21, CD19, and CD81) are stimulated by antigens or B cell epitopes (such as C3d) to activate B cells^15,16^. In the presence of MDA, immune tolerance complicates antigen presentation to T cells, the induction of a cellular immune response, and the activation of B cells through a T cell-dependent pathway. Thus, the B cells either activated directly through a dependent pathway, or continuously stimulated through the induction of a potent cellular immune response.

We previously developed an FMD vaccine strain with immune-enhancing effects that strengthened initial, intermediate, and long-term immunity through the simultaneous induction of cellular and humoral immunity, and presented an advanced vaccine platform using purified antigens derived from novel vaccine strain^17^. In the present study, we attempted to overcome MDA interference by directly stimulating the receptors on the B cell surface using the B cell epitope, C3d^18–20^. The specific epitope (13 amino acids) of C3d was inserted into an O PA2 or A22 VP1 backbone to create two FMD vaccine strains: O PA2-C3d (FMDV type O) and A22-C3d (FMDV type A). The immune-enhancing antigen purified from these vaccine strains was used to develop a novel FMD vaccine.

We investigated the ability of this vaccine to overcome MDA interference and induce an adaptive immune response in mice (experimental animal) and pigs (target animal).

## Results

### Development of the immune-enhancing FMD vaccine strain and the purification of inactivated antigens using O PA2-C3d and A22-C3d

**Fig. 1:**
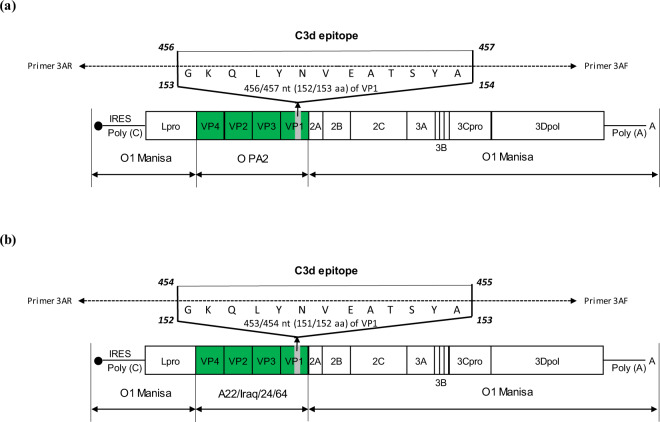
Construction of the immune-enhancing FMDV vaccine strains, O PA2-C3d and A22-C3d. (**a**, **b**) O PA2-C3d (**a**); A22-C3d (**b**). The B cell epitope, C3d (with 13 amino acid residues in the VP1 region) is used to prepare the virus. The O PA2 (O PA2-R) or A22 (A22-R) P1 strains—where the P1 region of O1 Manisa is substituted with O PA2 P1 or A22 P1—are used as the backbone to prepare the immune-enhancing FMD vaccine strains that can overcome interference by maternally-derived antibodies.

To develop FMD vaccine strains capable of overcoming MDA interference, capsid protein-coding sequence backbones based on the O1 Manisa-O PA2-R (O1 Manisa-O PA2) and O1 Manisa-A22/Iraq/24/64-R (O1 Manisa-A22) strains were used. We generated the FMD vaccine strains—FMDV type O and FMDV type A—by inserting the specific epitope of C3d (a B cell epitope) into the O PA2 and A22 VP1 coding region (Fig. 1a, b). The inactivated viral antigens were produced and purified using 2 types of immune-enhancing FMD vaccine strain (O PA2-C3d and A22-C3d) and 2 types of backbone viruses (O PA2 and A22) which were used as the positive control (PC). To better illustrate the properties of the antigen derived from O PA2-C3d and A22-C3d and confirm the formation of the SP band and the non-formation of the NSP band, the antigen was mounted on the antigen kit (PBM rapid test kit). Results confirmed structural proteins (SP) band formation even with a very small dose of 2.34 ng (1/640 dose) and no non-structural protein (NSP) band formation, indicating that the antigens were distinguishable from the field strains (Supplementary Figure 1a–d). The antigens (146 S particle) were purified through a sucrose gradient and observed under transmission electron microscopy (TEM), and formed the 146 S particle (Supplementary Figure 2a–d). In addition, we confirmed whether the FMDV, in which the specific epitope of ‘C3d’ was inserted, maintained the genetic stability of the virus even after passaging into cells, and finally verified no sequence change until the 4^th^ passage (Supplementary Figure 3). Therefore, we used this as the antigen for the FMD study vaccine.

### The immune-enhancing FMD antigen induces IFNγ secretion

To demonstrate the C3d-inserted FMDV antigen-induced specific cellular immune response, we confirmed the ‘C3d’ inserted FMDV (O PA2-C3d, A22-C3d) Ag-mediated IFNγ secretion via in vitro ELISpot assay using peritoneal exudate cells (PECs) isolated from mouse peritoneal lavage fluid and peripheral blood mononuclear cells (PBMCs) isolated from porcine whole blood. Inactivated FMDV antigen derived from O PA2-C3d and A22-C3d induced significantly higher IFNγ secretion than those in the control on murine PECs and porcine PBMCs (Supplementary Figure 4). Taken together, these results demonstrated that O PA2-C3d and A22-C3d can induce Th1-type immune responses.

### The immune-enhancing FMD vaccine exhibits high immunogenicity in mice and provides a strong host defense against FMDV infection

**Fig. 2:**
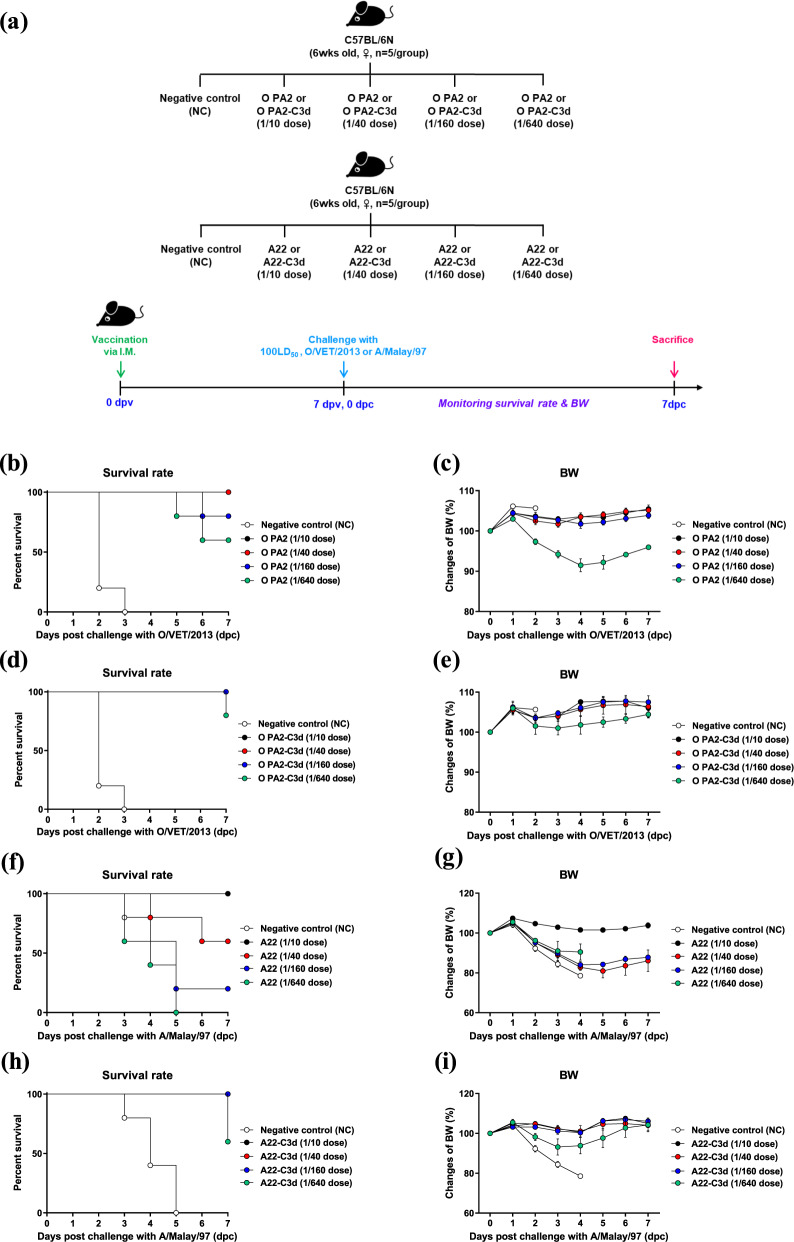
Vaccine efficacy and protective effects of O PA2-C3d and A22-C3d in mice. C57BL/6 mice (*n* = 5/group) were administered the test vaccine at 1/10, 1/40, 1/160, 1/640 doses of O PA2 or O PA2-C3d or A22 or A22-C3d antigen for cattle or pig use, ISA 206 (oil-based emulsion, 50%, w/w), 10% Al(OH)_3_, and 15 µg Quil-A. A negative control (NC) group was injected with the same volume of PBS. The test vaccines were injected intramuscularly into mice that were later challenged with FMDV type O (100 LD_50_ O/VET/2013) or FMDV type A (100 LD_50_ A/Malay/97) at 7 dpv. The survival rates and body weights were monitored for 7 dpc. (**a**–**i**) Experimental strategy (**a**); survival rates post-challenge with O/VET/2013 (**b**, **d**) or A/Malay/97 (**f**, **h**); changes in body weight post-challenge with O/VET/2013 (**c**, **e**) or A/Malay/97 (**g**, **i**). The data represent the mean ± SEM of triplicate measurements (*n* = 5/group).

To confirm the immunogenicity of the antigens derived from O PA2-C3d and A22-C3d, assess their potential as a master seed virus (MSV) for the FMD vaccine, and evaluate their protective effects against FMDV infection, experiments were performed according to the strategies illustrated in Fig. 2a.

The vaccine containing the backbone strain O PA2 derived antigen showed an immunogenicity of 55.72 protective dose 50 (50% protective dose; PD_50_) value (log_4_; four-fold dilution) as the mouse dose (one-tenth that for pigs) (Fig. 2b), and a body weight loss of approximately 10% at 1/640 dose (Fig. 2c). In contrast, the vaccine containing O PA2-C3d antigen showed 100% survival at the 1/10, 1/40, and 1/160 doses and 80% survival for the 1/640 dose with a 97.01 PD_50_ (log_4_) (Fig. 2d). There were almost no changes in body weight for the 1/10, 1/40, and 1/160 doses (Fig. 2e). The vaccine containing the backbone strain A22 antigen showed an immunogenicity of 6.06 PD_50_ (log_4_) (Fig. 2f), and showed a weight loss of approximately 20% at the 1/40–1/60 dose (Fig. 2g). However, the vaccine containing the A22-C3d antigen showed 100% survival for the 1/10, 1/40, and 1/160 doses and 60% survival for the 1/640 dose with 73.52 PD_50_ (log_4_) (Fig. 2h). There were no changes in body weight for the 1/10, 1/40, and 1/160 doses (Fig. 2i).

**Fig. 3:**
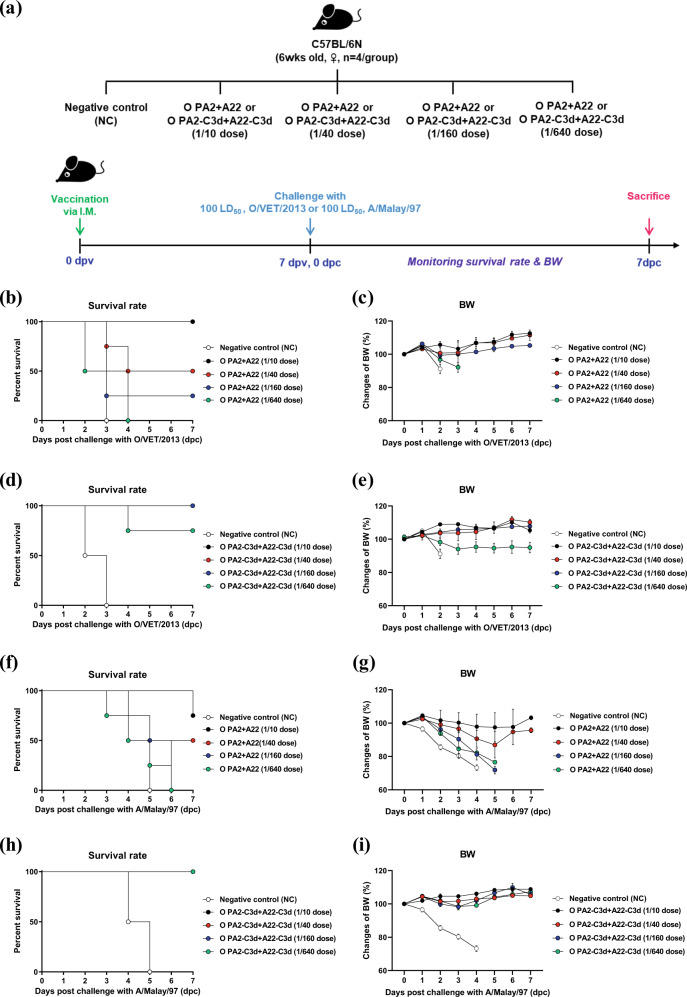
Vaccine efficacy and protective effects of a bivalent test vaccine containing the O PA2-C3d and A22-C3d antigens. C57BL/6 mice (*n* = 4/group) were administered the test vaccine at 1/10, 1/40, 1/160, 1/640 doses of O PA2 + A22 antigen or O PA2-C3d + A22-C3d antigen for cattle or pig use, ISA 206 (oil-based emulsion, 50%, w/w), 10% Al(OH)_3_, and 15 µg Quil-A. A negative control (NC) group was injected with the same volume of PBS. The test vaccines were injected intramuscularly into mice that were later challenged with FMDV type O (100 LD_50_ O/VET/2013) or FMDV type A (100 LD_50_ A/Malay/97) at 7 dpv. The survival rates and body weights were monitored for 7 dpc. (**a**–**i**) Experimental strategy (**a**); survival rates post-challenge with O/VET/2013 (**b**, **d**) or A/Malay/97 (**f**, **h**); changes in body weight post-challenge with O/VET/2013 (**c**, **e**) or A/Malay/97 (**g**, **i**). The data represent the mean ± SEM of triplicate measurements (*n* = 4/group).

To verify the immunogenicity of the bivalent vaccine (containing the O PA2-C3d + A22-C3d antigens) in mice, we conducted a PD_50_ test (Fig. 3a) and compared the results to those of the group that received the vaccine (containing the O PA2 + A22 antigens) as the backbone of the immune-enhancing vaccine strain (Fig. 3b–i). The bivalent vaccine containing O PA2 + A22 antigens showed PD_50_ (log_4_) values of 5.66 and 4 when challenged with O/VET/2013 (Fig. 3b, c) and A/Malay/97 (Fig. 3f, g), respectively, in mice. The bivalent vaccine containing O PA2-C3d + A22-C3d antigens showed high immunogenicity, with PD_50_ (log_4_) values of 90.5 and >128 when challenged with O/VET/2013 (Fig. 3d, e) and A/Malay/97 (Fig. 3h, i), respectively.

### The immune-enhancing FMD vaccine strain overcomes MDA interference, induces a potent adaptive immune response in pigs, and effectively maintains early, intermediate, and long-term immunity

**Fig. 4:**
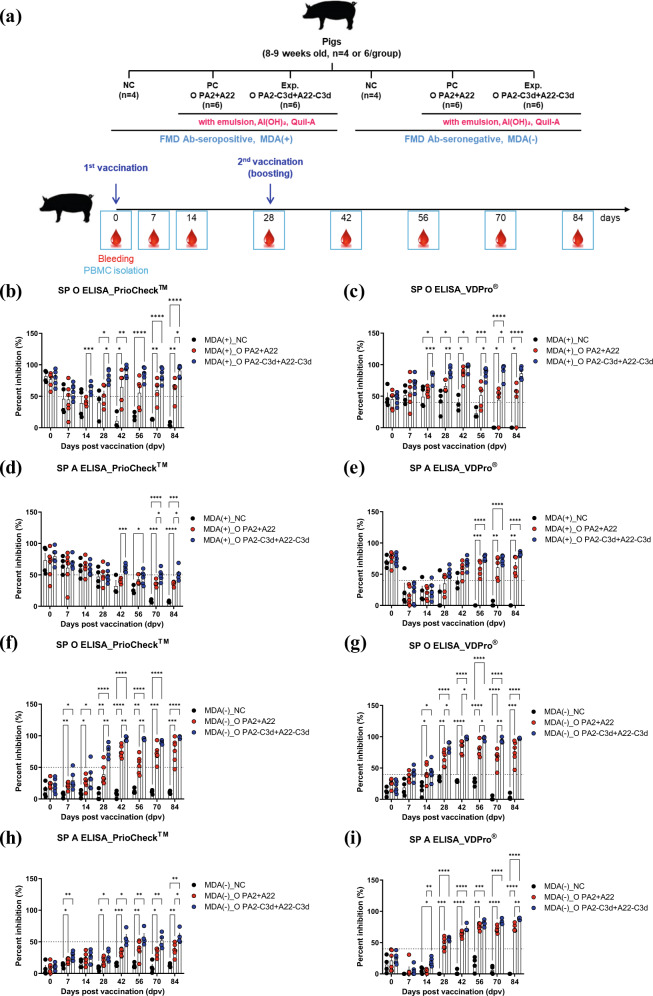
Immune responses mediated by the immune-enhancing FMDV (O PA2-C3d and A22-C3d), as measured by SP O and SP A ELISA for overcoming interference by maternally-derived antibodies (MDA) in pigs. Pigs (8–9 weeks old) that were FMD antibody-seropositive (MDA(+), *n* = 16) or FMD antibody-seronegative (MDA(−), *n* = 16) animals were divided into three groups, respectively: a negative control group (NC, *n* = 4/group), a positive control group (PC, *n* = 6/group), and an experimental group (Exp., *n* = 6/group). The Exp. group were administered the test vaccines containing 15 μg (1 dose for cattle and pig use) O PA2-C3d + A22-C3d antigen with ISA 206 (oil-based emulsion, 50%, w/w), 10% Al(OH)_3_, and 150 μg Quil-A. The positive control group received 15 μg (1 dose for cattle and pig use) O PA2 + A22 antigen with ISA 206 (oil-based emulsion, 50%, w/w), 10% Al(OH)_3_, and 150 μg Quil-A. A negative control (NC) group was injected with the same volume of PBS. The vaccination was performed twice at 28-day intervals, with 1 mL vaccine (1 dose) injected via a deep intramuscular route on the animals’ necks. Blood samples were collected at 0, 7, 14, 28, 42, 56, 70, and 84 days post vaccination in pigs for serological assays. (**a**–**i**) Study strategy (**a**); SP O antibody titers (PrioCheck^TM^ kit) in MDA(+) pigs (**b**); SP O antibody titers (VDPro^®^ kit) in MDA(+) pigs (**c**); SP A antibody titers (PrioCheck^TM^ kit) in MDA(+) pigs (**d**); SP A antibody titers (VDPro^®^ kit) in MDA(+) pigs (**e**); SP O antibody titers (PrioCheck^TM^ kit) in MDA(−) pigs (**f**); SP O antibody titers (VDPro^®^ kit) in MDA(−) pigs (**g**); SP A antibody titers (PrioCheck^TM^ kit) in MDA(−) pigs (**h**); SP A antibody titers (VDPro^®^ kit) in MDA(−) pigs (**i**). The data represent the mean ± SEM of triplicate measurements (*n* = 4 or 6/group). Statistical analyses were performed using a two-way ANOVA followed by Tukey’s test. ^*^p < 0.05; ^**^p < 0.01; ^***^p < 0.001; and ^****^p < 0.001.

To assess the immunogenicity of the antigens derived from the immune-enhancing FMD vaccine strains, O PA2-C3d and A22-C3d, we performed an target animal experiment using MDA-positive (MDA(+), FMD-seropositive) and -negative (MDA(−), FMD-seronegative) pigs, and evaluated their efficacy to induce an adaptive immune response and overcome MDA interference (Fig. 4a).

When the vaccine containing the O PA2-C3d + A22-C3d antigens was administered to MDA(+) pigs, the antibody titer (measured using SP O ELISA) significantly increased compared to that in the PC group vaccinated with the vaccine containing the O PA2 + A22 antigens at 14 dpv (*p* < 0.001, PrioCheck^TM^ and VDPro^®^ kits). The significant difference lasted until 28 dpv at *p* < 0.05 (PrioCheck^TM^ kit) and *p* < 0.01 (VDPro^®^ kit). After boosting the vaccine on 28 dpv, the antibody titer was significantly increased in the experimental group, whereas the NC group showed a gradual decrease in the antibody titer (Fig. 4b, c). The antibody titer (as measured by SP A ELISA) was higher in the experimental group than in the PC group at 42, 70, and 84 dpv (*p* < 0.001, *p* < 0.05, PrioCheck^TM^ kit) (Fig. 4d, e).

The vaccine was also administered to MDA(−) pigs to confirm the induction of adaptive immune response as well as intermediate and long-term immunity. The experimental group showed a significantly higher antibody titer (measured using SP O ELISA) from 28 dpv compared with the PC group (*p* < 0.01, PrioCheck^TM^ kit; *p* < 0.05, VDPro^®^ kit), with the titers remaining high at 42 to 70 dpv (*p* < 0.01 and *p* < 0.05) (Fig. 4f, g). The experimental group showed persistently higher level of the antibody titer (measured using SP A ELISA) in the MDA(−) group compared with the PC group, and significant differences were found between the groups at 14 dpv (*p* < 0.01, VDPro^®^ kit), and 84 dpv (*p* < 0.05, PrioCheck^TM^ kit; *p* < 0.01, VDPro^®^ kit) (Fig. 4h, i).

**Fig. 5:**
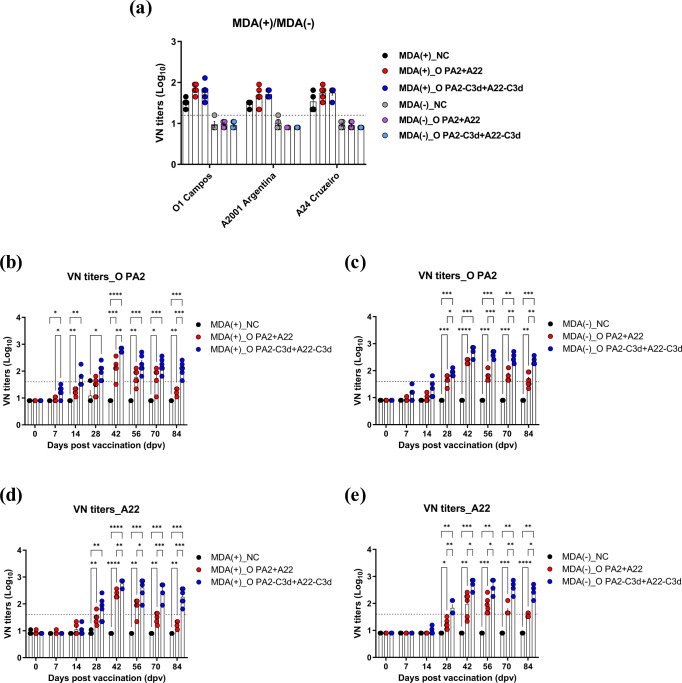
Immune responses mediated by the immune-enhancing FMDV (O PA2-C3d and A22-C3d), as measured by VN titers for overcoming interference of maternally-derived antibodies (MDA) in pigs. Pigs (8–9 weeks old) that were FMD antibody-seropositive (MDA(+), *n* = 16) or FMD antibody-seronegative (MDA(−), *n* = 16) animals were divided into three groups, respectively: a negative control group (NC, *n* = 4/group), a positive control group (PC, *n* = 6/group), and an experimental group (Exp., *n* = 6/group). The Exp. group were administered the test vaccines containing 15 μg (1 dose for cattle and pig use) O PA2-C3d + A22-C3d antigen with ISA 206 (oil-based emulsion, 50%, w/w), 10% Al(OH)_3_, and 150 μg Quil-A. The positive control group received 15 μg (1 dose for cattle and pig use) O PA2 + A22 antigen with ISA 206 (oil-based emulsion, 50%, w/w), 10% Al(OH)_3_, and 150 μg Quil-A. A negative control (NC) group was injected with the same volume of PBS. The vaccination was performed twice at 28-day intervals, with 1 mL vaccine (1 dose) injected via a deep intramuscular route on the animals’ necks. Blood samples were collected at 0, 7, 14, 28, 42, 56, 70, and 84 days post vaccination in pigs for serological assays. (**a**–**e**) O1 Campos, A2001 Argentina, and A24 Cruzeiro VN titers in MDA(+) or MDA(−) pigs (**a**); O PA2 VN titers in MDA(+) pigs (**b**); O PA2 VN titers in MDA(−) pigs (**c**); A22 VN titers in MDA(+) pigs (**d)**; A22 VN titers in MDA(−) pigs (**e**). The data represent the mean ± SEM of triplicate measurements (*n* = 4 or 6/group). Statistical analyses were performed using two-way ANOVA followed by Tukey’s test. ^*^p < 0.05; ^**^p < 0.01; ^***^p < 0.001; and ^****^p < 0.001.

The titers of neutralizing antibodies against O1 Campos, A2001 Argentina, and A24 Cruzeiro were measured in the MDA(+) and MDA(−) (MDA(+)/MDA(−)) pigs prior to vaccination (0 dpv) (Fig. 5a) and confirmed using a homologous virus for the O PA2 and A22 antigens in the PC group (Fig. 5b–e). The titer was >1.65 log_10_ and <1.2 log_10_ in the MDA(+) and MDA(−) groups, respectively. The antibody titer (as measured by SP O and SP A ELISAs) was high in the MDA(+) group at 0 dpv; however, the neutralizing antibody titer for O PA2 and A22 was below the protective level (<1.2 log_10_).

Compared to the NC and PC groups, the VN titer for O PA2 (Fig. 5b, c) was significantly higher in the experimental group administered the vaccine containing the O PA2-C3d + A22-C3d antigens from 7 dpv (NC *vs* Exp. *p* < 0.05; PC *vs* Exp. *p* < 0.05, MDA(+) pigs). The titer continuously increased until 28 dpv (NC *vs* Exp. *p* < 0.01, MDA(+) pigs; NC *vs* Exp. *p* < 0.001, PC *vs* Exp. *p* < 0.05, MDA(−) pigs). After boosting, the VN titer in the experimental group was higher than that in the PC group at 42 and 84 dpv (*p* < 0.01 and *p* < 0.001, respectively) on MDA(+) pigs, and 56, 70 and 84 dpv (*p* < 0.001, *p* < 0.01 and *p* < 0.01, respectively) on MDA(−) pigs.

The VN titer against A22 (Fig. 5d, e) was higher in the experimental group than in the NC and PC groups at 28 dpv (NC vs Exp. *p* < 0.01, MDA(+) pigs; NC *vs* Exp. *p* < 0.01, PC *vs* Exp. *p* < 0.01). A difference between the experimental and PC groups was observed at 42 and 84 dpv (*p* < 0.01, *p* < 0.05, *p* < 0.001 and *p* < 0.001, respectively, MDA(+) pigs; *p* < 0.05, *p* < 0.05, *p* < 0.01 and *p* < 0.05, respectively, MDA(−) pigs).

To evaluate the effect of the immune-enhancing vaccine strain, O PA2-C3d and A22-C3d on porcine total IgG, IgM, and IgA production in MDA(+)/MDA(−) pigs, we performed an immunoassay using sera from vaccinated pigs as shown in Fig. 4a. The concentration of IgG, IgM, and IgA were all significantly higher in MDA(+) pigs than in MDA(−) pigs at 0 dpv (*p* < 0.0001, *p* < 0.001, and *p* < 0.01. respectively). However, IgG, IgM, and IgA concentrations of MDA(+)/MDA(−) pigs were significantly higher in the experimental group than in the PC and NC group at 84 dpv after vaccination (*p* < 0.0001, *p* < 0.01, and *p* < 0.05, respectively).

**Fig. 6:**
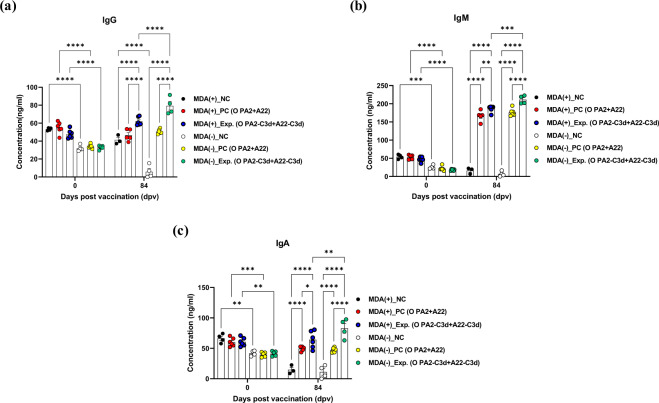
Immune responses mediated by the immune-enhancing FMDV (O PA2-C3d and A22-C3d), as measured by immunoglobulin subtypes such as IgG, IgM, and IgA in pigs. Pigs (8–9 weeks old) that were FMD antibody-seropositive (MDA(+), *n* = 16) or FMD antibody-seronegative (MDA(−), *n* = 16) animals were divided into three groups, respectively: a negative control group (NC, *n* = 4/group), a positive control group (PC, *n* = 6/group), and an experimental group (Exp., *n* = 6/group). The Exp. group were administered the test vaccines containing 15 μg (1 dose for cattle and pig use) O PA2-C3d + A22-C3d antigen with ISA 206 (oil-based emulsion, 50%, w/w), 10% Al(OH)_3_, and 150 μg Quil-A. The positive control group received 15 μg (1 dose for cattle and pig use) O PA2 + A22 antigen with ISA 206 (oil-based emulsion, 50%, w/w), 10% Al(OH)_3_, and 150 μg Quil-A. A negative control (NC) group was injected with the same volume of PBS. The vaccination was performed twice at 28-day intervals, with 1 mL vaccine (1 dose) injected via a deep intramuscular route on the animals’ necks. Blood samples were collected at 0, 7, 14, 28, 42, 56, 70, and 84 days post vaccination in pigs for serological assays. (**a–c**) IgG concentration (**a**); IgM concentration (**b**); IgA concentration (**c**). The data represent the mean ± SEM of triplicate measurements (*n* = 4 or 6/group). Statistical analyses were performed using two-way ANOVA followed by Tukey’s test. ^*^p < 0.05; ^**^p < 0.01; ^***^p < 0.001; and ^****^p < 0.001.

Notably, the concentration of these total immunoglobulin subtypes was significantly higher in MDA(−) pigs than in MDA(+) pigs by administration of a vaccine containing an immune-enhancing vaccine strain antigen (*p* < 0.0001, *p* < 0.001, *p* < 0.01, respectively) (Fig. 6a–c).

### The immune-enhancing FMD vaccine strain induces a robust cellular immune response by effectively inducing the expression of cytokines and co-stimulatory molecules in porcine peripheral blood mononuclear cells

**Fig. 7:**
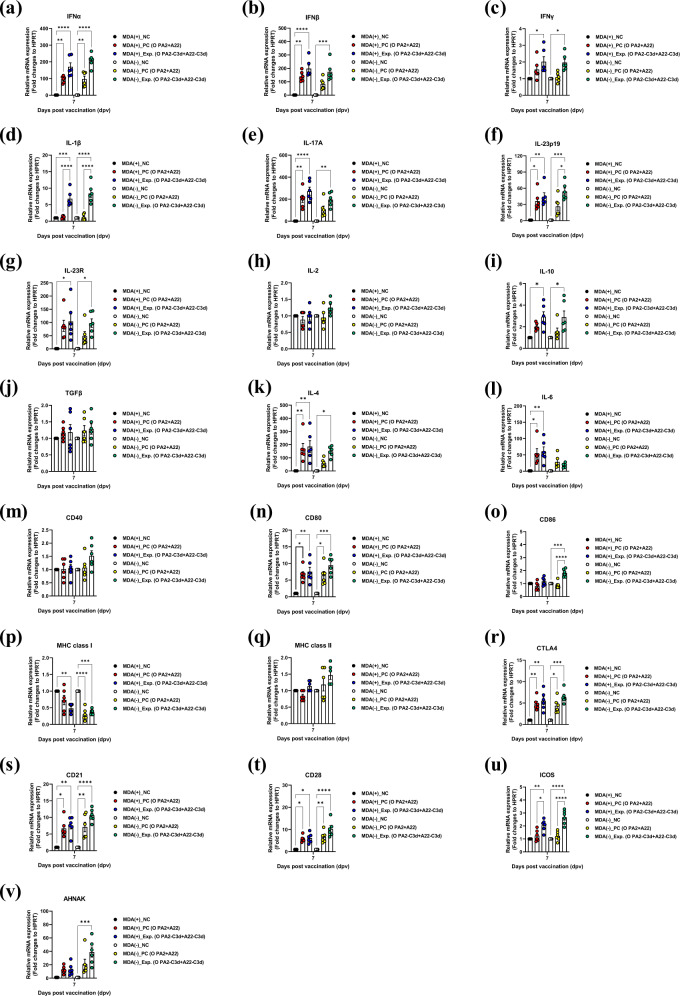
O PA2-C3d and A22-C3d induced the gene expression of cytokine and co-stimulatory molecules in porcine peripheral blood mononuclear cells. Porcine peripheral blood mononuclear cells (PBMCs) isolated from the whole blood of vaccinated pigs (*n* = 5/group) as described in Fig. 4a were used for qRT-PCR assays. Gene expression levels were normalized to HPRT levels and are presented as a relative ratio compared to control levels. (**a–v**) Gene expression levels of IFNα (**a**); IFNβ (**b**); IFNγ (**c**); IL-1β (**d**); IL-17A (**e**); IL-23p19 (**f**); IL-23R (**g**); IL-2 (**h**); IL-10 (**i**); TGFβ (**j**); IL-4 (**k**); IL-6 (**l**); CD40 (**m**); CD80 (**n**); CD86 (**o**); MHC class I (**p**); MHC class II (**q**); CTLA4 (**r**); CD21 (**s**); CD28 (**t**); ICOS (**u**); AHNAK (**v**). Statistical analyses were performed using one-way ANOVA followed by Tukey’s test. ^*^p < 0.05; ^**^p < 0.01; ^***^p < 0.001; and ^****^p < 0.001.

To determine the effect and mechanism of inducing immune-enhancing vaccine strain-mediated cellular immune response, we conducted qRT-PCRs using isolated porcine peripheral blood mononuclear cells (PBMCs) from the whole blood at each sampling time point in the vaccinated pigs with the vaccine containing the O PA2-C3d and A22-C3d antigens (Fig. 7a–v) as illustrated in Fig. 4a. The results showed changes in the gene expression of cytokines (IFNα, IFNβ, IFNγ, IL-1β, IL-17A, IL-23p19, IL-23R, IL-2, IL-10, TGFβ, IL-4, IL-6) and co-stimulatory molecules (CD40, CD80, CD86, MHC class I, MHC class II, CTLA4, CD21, CD28, ICOS, AHNAK) related to the induction of cellular immune response at 7 dpv.

The expression levels of genes encoding proinflammatory cytokines were very high in MDA(+)/MDA(−) pigs. In particular, the expression levels of Type I IFN such as IFNα and IFNβ were higher in the experimental group than in the NC group on 7 dpv in MDA(+)/MDA(−) pigs (*p* < 0.0001 and *p* < 0.001) (Fig. 7a, b). The expression level for IFNγ was somewhat lower than those for IFNα and IFNβ, but significantly higher in the experimental group than in the NC group in the MDA(+)/MDA(−) conditions (both *p* < 0.05) (Fig. 7c). IL-1β expression in the experimental group was markedly higher than in the NC group in the MDA(+)/MDA(−) conditions (*p* < 0.001 and *p* < 0.0001, respectively), and was significantly different from that in the PC group (both *p* < 0.0001) (Fig. 7d). IL-17A, IL-23p19 and IL-23R expressions were higher in the experimental group than in the NC group in the MDA(+)/MDA(−) conditions (IL-17A: *p* < 0.0001 and *p* < 0.01, respectively; IL-23p19: (*p* < 0.01 and *p* < 0.001, respectively; IL-23R: both *p* < 0.05) (Fig. 7e-g). Especially, IL-23p19 expression was significantly different between the experimental and PC groups in the MDA(−) condition (*p* < 0.05) (Fig. 7f). The expression levels of IL-2 and TGFβ were high in the order of experimental>PC > NC groups, although there were no statistically significant differences between the groups (Fig. 7h, j). Expression levels of the anti-inflammatory cytokine, IL-10, were higher in the experimental group than in the NC group in the MDA(+)/MDA(−) conditions (*p* < 0.05) (Fig. 7i). IL-4 and IL-6 expression levels were higher in MDA(+) pigs than in MDA(−) pigs, significantly different between the experimental and NC groups in the MDA(+) condition (*p* < 0.01). In the MDA(−) condition, IL-4 expression was significantly different between the experimental and NC groups (*p* < 0.05) (Fig. 7k, l).

The expression levels of the co-stimulatory molecules were lower compared to the overall expression levels of the cytokines, but were significantly higher in the experimental group than in the PC or NC groups. The expression levels of CD80 (Fig. 7n), CTLA4 (Fig. 7r), CD21 (Fig. 7s), CD28 (Fig. 7t), and ICOS (Fig. 7u) were significantly different between the experimental and NC groups in the MDA(+)/MDA(−) conditions (CD80: *p* < 0.01, *p* < 0.001, respectively; CTLA4: *p* < 0.01, *p* < 0.001, respectively; CD21: *p* < 0.01, *p* < 0.0001, respectively; CD28: *p* < 0.05, *p* < 0.0001, respectively; and ICOS: *p* < 0.05, *p* < 0.0001). Among these, CD80, CD21, and CD28 showed considerable changes in gene expression following the administration of the experimental vaccine. ICOS expression was higher in individuals vaccinated with the strain containing C3d than in those administered the backbone strain, and the difference was significant between the experimental and PC groups in the MDA(+)/MDA(−) conditions (*p* < 0.05 and *p* < 0.0001, respectively). Notably, expression levels of CD86 (Fig. 7o) and AHNAK (Fig. 7v) were significantly different between the experimental and NC groups in the MDA(−) condition (*p* < 0.0001 and *p* < 0.001, respectively). CD86 expression levels were also significantly different between the experimental and PC groups in the MDA(−) group (*p* < 0.0001), and MHC class I (Fig. 7p) genes showed lower expression levels in the MDA(−) condition than in MDA(+). The expression levels of CD40 (Fig. 7m) and MHC class II (Fig. 7q) genes were not significantly different between groups.

## Discussion

In the FMD vaccine, inactivated viral antigens are used. Several studies are also being conducted for vaccines using 1) a modified inactivated virus vaccine (DIVA vaccine)^21^, 2) viral vector vaccine^22^, 3) virus-like particle (VLP) vaccine^23^, 4) peptide vaccine^24^, 5) plant-based recombinant vaccine^25,26^, 6) DNA vaccine^27^, 7) RNA vaccine^28^, and 8) live-attenuated vaccine^29,30^. However, the inactivated viral vaccine remains superior in terms of efficacy. Recently, we developed an immune-enhancing vaccine strain in which the specific epitope of HSP70 (an immune-enhancing molecule) and 3 A (a T cell epitope) were inserted into the VP1 region (a viral surface structural protein) to increase the immunogenicity of the FMD viral antigen, elicit simultaneous induction of the cellular and humoral immune response, and strengthen the early, intermediate and long-term immunity^17^. Based on these strategies, we selected ‘C3d’ in this study to create a vaccine that could overcome the following limitations of the current commercially available FMD vaccine: MDA interference in young, week-old animals, and inhibition of the formation of active immunity upon vaccination.

Antibody-mediated immune responses orchestrated by B cells play an important role in protecting the host from pathogens. The signaling process is initiated by the recognition of the antigen by the highly specific B cell receptor (BCR) displayed on the B cell surface. Antigen affinity discrimination is an intrinsic function of the BCR and antigen with varying affinity for BCR induce differential B cell response^31,32^. The interaction between the antigen and BCR induces receptor micro-clustering on the cell surface. This results in a signaling cascade through calcium influx, causing transcriptional activation in B cells^33,34^. The signaling process through the BCR complex is tightly regulated by several different co-receptors such as stimulatory receptors (e.g., CD19, CD21), and inhibitory receptors (e.g., CD22, CD45, FcγRIIb)^35,36^. B cells are regulated through receptors on the surfaces of multiple checkpoints^37^, and strategically targeting the B cell receptors in each of these signals can help modulate the immune response to vaccination.

The complement system is essential for immune homeostasis and plays an important role in linking innate and adaptive immune responses. The most important member of the complement system is C3d—a B cell epitope derived from complement component 3 (C3)—which forms a C3d-tagged microorganism (pathogen) or antigen capable of binding to CR2 (CD21) in B cells and follicular dendritic cells (DCs) for opsonization^38,39^. When a C3d-tagged pathogen simultaneously interacts with CD21 and BCR, the pathway crosstalk reduces the antigen threshold required to stimulate the activation of B cells^40,41^. Inhibiting antigen presentation and apoptosis of the antigen itself helps induce and maintain memory^42,43^.

CD21 is a co-receptor commonly found on the surface of B cells along with CD19 and CD81 and plays an essential role in immune regulation. CD21 does not have a signaling motif, however, when bound to C3d, it generates a downstream signal through CD19 immunoreceptor tyrosine-based activation motifs, which improves the survival and proliferation of B cells^44^. Of all the B cell-targeting receptors, CD21 has been the most extensively researched for its use as an adjuvant^45^.

Previous studies have attempted to increase B cell signaling and induce both cellular and humoral immunity against various antigens using C3d—a molecular adjuvant that acts as a key interface between innate immunity and adaptive immunity^46–48^. Human C3d has an abnormally high frequency of T cell epitopes, and the C3d covalently bound to an antigen enhances the cellular immunity to antigens lacking a T cell epitope. This is due to receptor cross-linking for surface IgM between CD21 and antigens in naïve B cells^46,49^, which allows C3d to act as a strong fusion-adjuvant by donating the T cell epitope to the antigen up-taking cell when binds to the target antigen.

Based on these findings, we inserted the specific site of C3d into the VP1 region of FMDV type O (O PA2) and FMDV type A (A22) to induce potent adaptive immune responses by directly stimulating receptors on the surface of B cells (CD21 and BCR) and developed advanced FMD vaccine strains O PA2-C3d and A22-C3d that can overcome MDA interference (Fig. 1).

The purified antigen (146 s particle) was observed under the electronic microscope (TEM), and we determined that this antigen could be used as the FMD vaccine (Supplementary Figure 1, 2). The immune-enhancing vaccine stain, into which the C3d epitope was inserted, maintained genetic stability even after passage in cells, and these virus-derived-inactivated antigens induced the IFNγ secretion in murine PECs and porcine PBMCs to induce a cellular immune response (Supplementary Figure 3, 4). Before the target animals (pigs) experiment, mice were used to compare the immunogenicity (PD_50_ value) of the monovalent vaccines containing the O PA2-C3d or O PA2 or A22-C3d or A22 antigens and the bivalent vaccines containing O PA2-C3d + A22-C3d or O PA2 + A22 (as the backbone).

The efficacy of the novel FMD vaccine strain fused with C3d was superior to that of the backbone vaccine strain (Figs. 2, 3). Thus, O PA2-C3d and A22-C3d have excellent immunogenicity as immune-enhancing FMD vaccine strains and play a key role in the induction of short-term immunity and initial host defense. The bivalent vaccine containing the O PA2-C3d + A22-C3d antigens was more effective in overcoming MDA interference due to the simultaneous induction of robust cellular and humoral immune responses at the initial stage after vaccination.

Subsequently, an target animal experiment was conducted using MDA(+) and MDA(−) pigs to evaluate the vaccine’s efficacy to overcome MDA interference and simultaneously induce cellular and humoral immune responses. In the MDA(+) animals, the O PA2-C3d + A22-C3d antigens showed a strong efficacy to overcome MDA interference. The antibody titer somewhat decreased at the initial stage of vaccination but increased after boosting. This may be because the SP region is different from that of the antigen coated on the commercially available SP A ELISA plate, which could have led to low detection and affected the PI value. In contrast, in MDA(−) animals, O PA2-C3d + A22-C3d induced a significant increase in antibody titers compared to the O PA2 + A22 backbone (Fig. 4).

To confirm the virus-neutralizing effect, VN titers were measured for O PA2 and A22. The MDA(+) animals were derived from mothers (sows) with a history of vaccination with company B (the source of commercial vaccine is not listed to protect the company’s rights and interests as well as to avoid disputes). To identify for the presence of MDA before vaccination, the VN titers were measured for O1 Campos, A2001 Argentina, and A24 Cruzeiro using serum at 0 dpv. At the initial stage (0 dpv), regardless of the MDA(+)/MDA(−) status, the VN titers for O PA2 and A22 were log_10_ < 1.2, which is below the protective level.

Efficacy evaluation of FMD vaccine is determined using cattle rather than pigs, along with the evaluation criteria. In cattle, 1.2 log_10_ (16-fold neutralizing antibody titer) represents over 50% protection, while 1.65 log_10_ (45-fold neutralizing antibody titer) represents over 90% protection^50^. A previous study has suggested that FMD vaccine-induced host defense is possible at VN titer by vaccination of >2 log_10_^51^. According to the FMD vaccine evaluation criteria in Korea, host defense is possible when the VN titer induced by commercial vaccination is >1.65 (Log_10_).

The low VN titers for O PA2 and A22, regardless of MDA(+)/MDA(−) status, is attributed to the antigen in the vaccine strain used to vaccinate the sows differed from the one used here. Since the outbreak of large-scale FMD in Korea in 2010, prevention and control for domestic FMD-sensitive animal including pigs, cattle, sheep, and goat have been accomplished via vaccination. The government has mandated FMD vaccination for pigs and cattle aged 8 to 12 weeks on all farms, regularly verified the antibody titer using SP O, A ELISA, and imposed a fine if the FMD-specific antibody was negative or lower than a certain threshold. Therefore, it is difficult to obtain MDA-free FMD antibody-seronegative pigs from young individuals in Korea. Meanwhile, Korea is importing commercial FMD vaccine form Merial Co. Ltd. (Lyon, France), Biognésis Bagó Ltd. (Buenos Aires, Argentina) and FGBI “Arriah” (Vladimir, Russia). The vaccine is administered to sows from these three manufacturers, hence the MDA is also dependent on the antigen contained in the vaccine received. Commercial vaccines have a disadvantage because it is challenging to overcome MDA interference. Therefore, we confirmed whether MDA could be overcome under conditions similar to those of outdoor farms by administering with a test vaccine containing a vaccine strain with ‘C3d’ into sows already vaccinated with an FMD vaccine distributed in Korea.

In the MDA(+) and MDA(−) groups, the VN titers were significantly higher in groups administered the O PA2-C3d + A22-C3d bivalent vaccine than those administered the vaccine with O PA2 + A22 (Fig. 5).

Here, O PA2-C3d and A22-C3d were effectively induced not only the SP-specific antibody titers (via SP ELISA), but also the SP-nonspecific antibody including IgG (an indicator of neutralizing antibody), IgM (the first natural antibody induced during pathogen infection or vaccination) and IgA (the key player in inducing mucosal immunity) levels in MDA(+)/MDA(−) animals (Fig. 6). Maternal IgG and IgA can attenuate mucosal helper cell responses in early infancy^52^, and maternal IgG regulatory T cell epitope induce immune tolerance rather than immunogenicity^53^.

Based on the results above, O PA2-C3d and A22-C3d, the immune-enhancing FMD vaccine strains, can effectively induce active immunity in the host by overcoming MDA interference-mediated immune tolerance in MDA(+) animals, and may also induce a robust cellular and humoral immune response when vaccinated in MDA(−) animals or 2–3 months-old (which is the period when the MDA titers are lower according to the current vaccine program) animals. These results suggest that C3d spiking on antigens continuously stimulates B cell surface receptors to directly activate B cells and the highly immunogenic O PA2-C3d and A22-C3d antigens provide a potent T cell-mediated immune response resulting in more efficient high-titer antibodies and neutralizing antibodies.

To demonstrate this, we identified a bivalent vaccine-mediated cellular immune response in MDA(+)/MDA(−) pigs. The vaccine containing the O PA2-C3d + A22-C3d antigens increased the expression of type I IFN with a significant antiviral effect at the initial stage of vaccination (7 dpv) in both MDA(+)/MDA(−) conditions. Thus, it can provide effective protection against FMDV infection at the early stage of vaccination.

The expression of IFNγ—a T helper (Th) 1 cell-related cytokine—was lower than that of IFNα and IFNβ, but showed a ≥ 2 fold-change (*p* < 0.05), indicating that it could effectively induce a T cell-mediated cellular immune response. The expression levels of IL-1β and Th17 cells (involved in inflammasome activity) and of unconventional T cells (γδ T cells, invariant natural killer T (iNKT) cells, and mucosal-associated invariant T (MAIT) cells)-derived IL-17A were very high in the experimental group administered the antigen with C3d. Our previous study suggested that the expression of IL-23p19/IL-23R signaling is critical in the initial defense of the host, while in this study, their expressions were at the level of a ‘cytokine storm’.

When IL-23A is secreted through the stimulation of PRRs in innate immune cells (such as DCs and macrophages (Mϕs)), binds to IL-23R on the surface of unconventional T cells (innate-like immune cells), and stimulates the production of IL-17A. IL-17A plays a decisive role in the early stage of host defense by recruiting neutrophils to the pathogen infection site and forming neutrophil extracellular traps (NET) to induce NETosis of the pathogen^54,55^. In addition, the IL-23/IL-17 axis links innate and adaptive immunity^56^, and the vaccine containing the O PA2-C3d + A22-C3d antigen induced innate and adaptive immune responses simultaneously through the secretion of these proinflammatory cytokines.

TGFβ^57^ is involved in the development of IL-2^58^ and regulatory T cells (T_regs_), and essential for the generation of memory cells. However, TGFβ expression was slightly higher in the experimental group, but not significant at 7 dpv. The expression of IL-10 (an anti-inflammatory cytokine) was also significantly higher in the experimental group (*p* < 0.05), likely due to host homeostasis to control the ‘cytokine storm’ of inflammatory cytokines. The expression of the Th2 cell-derived cytokines, IL-4 and IL-6, was higher in the MDA(+) group than in the MDA(−) group.

These cytokine expression levels were increased by the initial stage of FMD vaccination in the presence of passive immunity by the MDA.

The CD80 and CD86 co-stimulatory signals promote T cell activation in cooperation with the T cell receptor (TCR) signal, and were increased in the O PA2-C3d + A22-C3d administration group. We believe that the immune-enhancing FMD vaccine strains stimulated T cells by effectively presenting the antigen. The gene expression of MHC class I was higher in the MDA(+) group than in the MDA(−) group, and was lower in the vaccinated group than in the NC group. This may be due to the inhibition of antigen recognition by cytotoxic CD8^+^ T cells at the initial stage of vaccination, as the gene expression was low in the presence of passive immunity. In contrast, the expression of MHC class II genes was higher in the MDA(−) group than in the MDA(+) group. Although there were no significant differences between the groups, there was an increasing tendency in the experimental group. This suggests that the C3d-containing vaccine strains activated the CD4^+^ T cells that induced cooperation and regulation of effector cells through MHC class II antigen-presenting cells (DCs, Mϕs, and B cells). We hypothesize that the interaction with the MHC complex can induce continuous cell-cell contact formation and T cell activation.

The expression of CD21—a direct C3d receptor—increased significantly in the MDA(+)/(MDA(−) conditions following vaccination with the C3d-containing vaccine, suggesting that B cell activation is possible via stimulation by C3d and the binding of CD21. CD28 and ICOS are co-stimulated during T cell activation and play an important role in the induction of memory T cells. Their expression levels were significantly increased in the MDA(+)/MDA(−) conditions following administration of the C3d-containing vaccine.

The C3d-containing vaccine may have increased the expression of IFNγ, thereby increasing the co-stimulation of T cells and lymphocytes.

In addition, ICOS acts as an immunoglobulin domain, is reported^59^ to have an effect on the intestinal immune network for IgA production, and may be used as an FMD vaccine strain for simultaneous induction of systemic and mucosal immunity in the future. CTLA4 expression also showed a similar pattern to that of ICOS, and the CTLA pathway may have been induced by the expression of ICOS. The induced CTLA4 in the cytoplasmic domain may have caused the conversion of T_reg_ cells to suppress autoimmunity due to the ‘cytokine storm’ of proinflammatory cytokines induced by FMD vaccination.

The expression of AHNAK was markedly increased in the MDA(−) group administered the O PA2-C3d + A22-C3d vaccine (*p* < 0.001). AHNAK is a large (700 kDa) structural scaffold protein^60^ involved in cellular processes including cell structure, intracellular trafficking, membrane repair^61^, regulated exocytosis^62^, T cell differentiation, and calcium signaling^63^ during T cell activation. Cytolytic CD8^+^ T cells (CTLs) kill virus-infected cells in a calcium-dependent manner. AHNAK is expressed in mature CTLs but not in naïve CD8^+^ T cells. Calcium entry is required for important for proper functioning of cells and for inducing an immune response. In fact, ANHAK-deficient (Ahnak1^−/−^) CTLs show a significant decrease in Granzyme B production, cytolytic activity, and IFNγ secretion after TCR stimulation^64^. Therefore, we suggest that the vaccine containing the O PA2-C3d + A22-C3d antigen can induce T cell activation and CTL response through the expression of AHNAK.

Collectively, this study suggests that a novel strategy vaccine containing the antigen derived from the C3d-fused FMD vaccine strain induces a robust cellular and humoral immune response at the early stage of vaccination, and can effectively overcome MDA interference through stimulation of B cell receptor. These results provide insights into developing the next-generation core source-based technology that enables vaccine-mediated active immunity.

## Methods

### Preparation of the recombinant plasmid

The recombinant plasmid was prepared as follows^17,65^. The whole FMD-O1 Manisa virus genome (GenBank Accession No. AY593823.1) was PCR-amplified and inserted into the pBluescript SK II (Agilent, Santa Clara, CA, USA) plasmid to produce the pO-Manisa plasmid. In the pO-Manisa plasmid, the gene encoding the structural protein was substituted with the gene encoding structural proteins from O-serotype FMDV O PA2 (GenBank Accession No. AY593829.1) or A-serotype FMDV A22/Iraq/24/64 (GenBank Accession No. AY593764.1) to prepare two types of plasmids: pOm-O PA2-P1 or pOm-A22-P1. For type O, O PA2 was determined as the strongest candidate vaccine strain. This was based on the results of a previous study which confirmed the vaccine matching rate in floating cells—especially the antigen-mediated immunogenicity in experimental animals (mice) and target animals (pigs). For type A, the vaccine matching rate tends to be low in terms of global incidence; however, A22 was suitable. The C3d (B cell epitope) sequence (5′-GGTAAGCAGCTCTACAACGTGGAGGCCACATCCTATGCC-3′, corresponding to the amino acid sequence, GKQLYNVEATSYA) was inserted in the VP1 protein coding sequence between the 456^th^ and 457^th^ base pairs (amino acid positions, 152 and 153) in PA2-C3d, and between the 453^rd^ and 454^th^ base pairs (amino acid positions, 151 and 152) in A22-C3d. Next, 300 ng/μL of pOm-A22-P1 (PCR template), 1 μL (10 pmol/μL) of the C3d F primer (5′-GGAGGCCACATCCTATGCCCGCGAGAGGCCCTAGGTCGC-3′), and 1 μL (10 pmol/μL) of the C3d R primer (5′-ACGTTGTAGAGCTGCTTACCGCGAGGGTCGCCGCTCAGCT-3′) were used to prepare the target plasmid using the same self-ligating method used in the previous study^17,65^. Figure 1a and b illustrates the schematic of the final plasmid for O PA2-C3d and A22-C3d, respectively. The PCR conditions were as follows: 10 μL of the 5X Phusion HF buffer (Thermo Scientific, Waltham, MA, USA), 1 μL of 10 mM dNTP (Invitrogen, Carlsbad, CA, USA), 1 μL of 2 U/μL Phusion DNA polymerase (Thermo Scientific), and 35 μL of sterile distilled water were subjected to 98 °C (30 s), followed by PCR amplification for 25 cycles at 98 °C (10 s), 65 °C (20 s), and 72 °C (2 min and 30 s), followed by a final cycle at 72 °C (10 min). Next, 1 μL of DpnІ (Enzynomics, Daejeon, Korea) was added to the 25 μL of PCR product and allowed to incubate at 37 °C for 1 h. Next, 35 μL of sterile distilled water, 5 μL of Ligation High (TOYOBO, Osaka, Japan), and 1 μL of 5 U/μL T4 polynucelotide kinase (TOYOBO) were added to 4 μL of the DpnI-treated product. The mixture was ligated in a 16 °C water bath for 1 h, following which the plasmid was transformed into 100 μL of DH5α cells (Yeastern Biotech, Taipei, Taiwan) according to the manufacturer’s protocol. The transformed cells were smeared onto an agar plate containing ampicillin and incubated overnight at 37 °C. A colony was picked from the plate with a pipette tip and mixed with 18 μL of sterile distilled water, 1 μL (10 pmol/μL) of a universal forward primer for VP1 (5′-AGNGCNGGNAARTTTGA-3′), and 1 μL (10 pmol/μL) of a universal reverse primer for VP1 (5′-CATGTCNTCCATCTGGTT-3′) in a colony PCR tube. This mixture was subjected to 94 °C (5 min), followed by PCR amplification for 25 cycles at 94 °C (30 s), 55 °C (30 s), and 72 °C (1 min), followed by a final cycle at 72 °C (5 min). In the aforementioned universal primer, N can represent any nucleotide. Next, 5 μL of the PCR sample was mixed with 1 μL of 6X loading buffer (DYNE BIO, Gyeonggi, Korea) before being loaded onto an agarose gel alongside 5 μL of 100 bp marker (DYNE BIO). After electrophoresis at 100 V (30 min), the bands were assessed on a Gel Doc (Bio-Rad, Hercules, CA, USA) system.

Next, 5 μL of PCR product was mixed with 2 μL of ExoSAP (Thermo Scientific) and PCR amplified at 37 °C (15 min) and 85 °C (15 min). The insertion of the epitopes into the VP1 sequence was confirmed via full DNA sequencing. Next, the colony was placed in 200 mL of LB media containing ampicillin and incubated overnight at 37 °C with shaking. The midi-prep method (Macherey-Nagel, Duren, Germany) was used to prepare the plasmid^17^.

### Preparation of the immunostimulating recombinant FMD vaccine strain

The recombinant FMD virus was recovered by transfecting BHKT7-9 (a cell line that expresses T7 RNA polymerase) with the recombinant plasmid prepared above using the Lipofectamine 3000 reagent (Invitrogen), followed by incubation for 2–3 days. The prepared virus was passaged in fetal goat tongue (ZZ-R) cells or baby hamster kidney-21 (BHK-21) cells for viral proliferation^17^.

### Purification of the antigen from recombinant FMDV type O and type A presenting C3d-epitopes

The purified antigen (inactivated virus) was prepared in BHK-21 cells infected with the recombinant immunostimulatory FMDV O PA2-C3d and A22-C3d constructed for the swift phenotype of VP1 (referred sequence) by reverse genetics according to the method described by Lee et al., with modifications^17^. For viral infection, the culture medium was replaced with serum-free Dulbecco’s modified Eagle’s medium (HyClone, Logan, UT, USA), and the cells were inoculated with the virus by incubating for 1 h at 37 °C in a 5% CO_2_ atmosphere. The extracellular viruses were then removed. Twenty-four hours post-infection, the viruses were inactivated by two treatments of 0.003 N binary ethylenimine for 24 h in a shaking incubator, followed by concentration with polyethylene glycol (PEG) 6000 (Sigma-Aldrich, St. Louis, MO, USA)^66^.

The virus concentrate was layered on 15–45% sucrose density gradients and centrifuged. After ultracentrifugation, the bottom of the centrifuge tube was punctured and 1 mL fractions were collected. The presence of FMDV particles was confirmed in a sample of each fraction by performing optical density measurements using a lateral flow device (BioSign FMDV Ag; Princeton BioMeditech, Princeton, NJ, USA). Prior to use in field experiments, the pre-PEG treated supernatant was passage through ZZ-R and BHK-21 cells at least twice to ensure that no cytopathic effects (CPE) occurred, thereby confirming the absence of any live virus in the supernatant.

### Confirmation of structural and non-structural proteins using purified antigens and examination of 146 S particles using TEM

The SPs of purified antigen expression were confirmed in cells infected with immunopotent recombinant FMDV O PA2-C3d, A22-C3d, O PA2 and A22 using rapid antigen kits (PBM kit, PBM Co Ltd., Princeton, NJ, USA). The results showed band formation for the SPs and no band formation for the NSPs of FMDV. The virus particle (146 S) was characterized by TEM imaging^17^.

### Mice

The animal protocol was conducted according to the method described by Lee et al. and Jo et al.^17,67^. Age- and sex-matched wild-type C57BL/6 mice (females, 6–7 weeks old) were purchased from KOSA BIO Inc. (Gyeonggi, Korea). All mice were housed in microisolator cages with *ad libitum* access to food and water in a specific pathogen-free biosafety level 3 animal facility at the Animal and Plant Quarantine Agency. All animals were allowed to adapt for at least one week before use in experiments. The housing room was set to a 12 h:12 h light/dark cycle, a temperature of approximately 22 °C, and relative humidity of approximately 50%. The studies were performed according to institutional guidelines and approved by the Ethics Committee of the Animal and Plant Quarantine Agency (accreditation number: IACUC-2021-584).

### PECs isolation and cell culture

Naïve mice were anesthetized using CO_2_ and sacrificed. The peritoneal cavities were lavage with 5 mL of chilled Hank’s balanced salt solution (HBSS, Gibco, Waltham, MA, USA) buffer without Ca^2+^/Mg^2+^/phenol-red. The peritoneal lavage fluid was centrifuged at 300 × *g* for 10 min at 4 °C. The pelleted PECs were resuspended and counted using a Bio-Rad TC20 Automated Cell Counter (Bio-Rad). All cells were freshly isolated before use. No cryopreserved cells were used in any experiment. Purified PECs were then cultured in a complete medium consisting of Roswell Park Memorial Institute (RPMI) 1640 (Gibco, Carlsbad, CA, USA) supplemented with 10% fetal calf serum (HyClone), 3 mM L-glutamine (Sigma-Aldrich), 10 mM HEPES (Sigma-Aldrich), 100 U/mL penicillin/streptomycin (Sigma-Aldrich), and 0.05 mM 2-beta-mercaptoethanol (Sigma-Aldrich). Incubations were carried out at 37 °C and 5% CO_2_.

### PBMCs isolation and cell culture

Porcine PBMCs were isolated from whole blood of FMD antibody-seronegative pigs as donors (8-9 weeks old animals, n = 3/group) according to the method described by Lee et al. and Jo et al.^17,67^. Whole blood (20 mL/donor) was independently collected in BD Vacutainer heparin tubes (BD, Becton, Dickinson and Company, Franklin Lakes, NJ, USA). PBMCs were isolated using Ficoll-Paque PLUS (GE Healthcare Bio-Sciences Corp., Piscataway, NJ, USA) gradient centrifugation. Residual red blood cells were lysed with ammonium–chloride–potassium (ACK) lysing buffer (Gibco). The PBMCs were suspended in Ca^2+^/Mg^2+^-free DPBS (Gibco) and counted using a Bio-Rad TC20 Automated Cell Counter (Bio-Rad). All cells were freshly isolated before use. No cryopreserved cells were used in any experiment. Purified PBMCs were then resuspended in RPMI-1640 (Gibco) medium supplemented with 10% FBS (Gibco), 3 mM L-glutamine (Sigma-Aldrich), 10 mM HEPES (Sigma-Aldrich), and 100 U/mL penicillin–streptomycin (Sigma-Aldrich). Incubations were carried out at 37 °C and 5% CO_2_.

### Antigen-induced IFNγ ELISpot assay on PECs and PBMCs in vitro

O PA2-C3d and A22-C3d antigen-mediated IFNγ secretion was analyzed using commercial ELISpot assay kits (catalog no. EL485 and EL985 for mouse and porcine, respectively; R&D Systems, Minneapolis, MN, USA) according to the manufacturer’s instructions. Briefly, isolated murine PECs or porcine PBMCs (5 × 10^5^ cells/well) were cultured in a 96-well PVDF-backed microplates containing a monoclonal capture antibody specific for mouse or porcine IFNγ and stimulated with 4 μg/mL (Final concentration) of inactivated FMDV (O PA2, O PA2-C3d, A22, A22-C3d) antigen at each concentration for 18 h in a humidified incubator at 37 °C with 5% CO_2_. As negative and positive control, PBS and 5 μg/mL of phorbol myristate acetate (PMA, Sigma-Aldrich) were used, respectively. The plates were washed with wash buffer and incubated with biotinylated anti-mouse IFNγ antibodies (1:119) or anti-porcine antibodies (1:119) overnight at 4 °C, followed by AP-conjugated streptavidin (1:119) at RT for 2 h. The plates were washed, developed with 5-Bromo-4-Chloro-3’ Indolyphosphate p-Toluidine Salt (BCIP)/Nitro Blue Tetrazolium Chloride (NBT), and counted using an ImmunoSpot ELISpot reader (AID iSpot Reader System; Autoimmune Diagnostika GmbH, Strassberg, Germany). The results were presented as spot forming unit (SFU).

### Evaluation of immunogenicity in experimental animals (mice) vaccinated with the immune-enhancing FMD vaccine strains, O PA2-C3d and A22-C3d

The animal protocol was conducted according to the method described in Lee et al. and Jo et al.^17,67^ as mentioned in the *Mice* of the method section.

To validate the immunogenicity and short-term immunity of purified antigens isolated from immunopotent FMDV O PA2-C3d and A22-C3d, and to verify their potential as a master seed virus for the development of an FMD vaccine, we conducted animal experiments as follows. The vaccine compositions used in the experiments were as follows: purified antigens isolated from O PA2-C3d and A22-C3d (15 μg/dose/mL; 1/10–1/640 of the dose for pigs), ISA 206 (Seppic, Paris, France; 50% w/w), 10% Al(OH)_3_, and 15 μg/mouse Quil-A (InvivoGen, San Diego, CA, USA). Mice were vaccinated by I.M. injection in the thigh muscle (0 dpv) and challenged with FMDV (100 LD_50_ of O/VET/2013, ME-SA topotype or 100 LD_50_ A/Malay/97, SEA topotype) by I.P. injection at 7 dpv. Mice in the NC group received an equal volume of PBS (pH 7.0) administered via the same route. Survival rates and changes in body weight were monitored for up to 7 dpc to assess short-term immunogenicity (Fig. 2a). The PD_50_ test was conducted as a preliminary experiment to verify the immunogenicity of the bivalent study vaccine (containing the O PA2-C3d + A22-C3d antigens) in pigs (Fig. 3a). The results were compared to those of the group that received the study vaccine (containing the O PA2 + A22 antigens) used as the backbone of the immune-enhancing vaccine strain. The vaccine compositions used in the experiment were as follows; O PA2-C3d + A22-C3d antigens (15 μg + 15 μg/dose/mL, 1/10–1/640 dose) or O PA2 + A22 antigens (15 μg + 15 μg/dose/mL, 1/10–1/640 dose), ISA 206 (50%, w/w), 10% Al(OH)_3_, and 15 μg Quil-A/mouse. Animals in the NC group were administered the same volume of PBS by the same route. In mice, vaccination was administered I.M. on 0 dpv, and FMDV (100 LD_50_ of O/VET/2013, ME-SA topotype or 100 LD_50_ of A/Malay/97, SEA topotype) was administered I.P. at 7 dpv. Survival and changes in body weight were monitored until 7 dpc.

### Evaluation of immunogenicity in pigs vaccinated with the immune-enhancing FMD vaccine strains, O PA2-C3d and A22-C3d

To evaluate the potential of O PA2-C3d and A22-C3d as an FMDV vaccine strain and to investigate its ability to induce cellular and humoral immune responses and long-term immunity, preliminary experiments were conducted using pigs according to the method described by Lee et al. and Jo et al.^17,67^. The pigs (8–9 weeks old; n = 32) were screened based on antibody titers (PI value: 50%) using the ELISA tests for SP O and SP A, and VN titers (1.65 log_10_), and were classified as MDA(+) and MDA(−) (n = 16 per group). In each group, the pigs were further divided into 3 groups: NC (negative control), O PA2 + A22-treated (positive control, PC), and O PA2-C3d + A22-C3d-treated. The animals were randomly divided into three groups (n = 5/group) (Fig. 4a). The animals were isolated in closed ABSL3 containments during the study, provided with *ad libitum* access to food and water, and used for the experiment after at least one week of adaptation. The housing room was set to a 12 h:12 h light/dark cycle, a temperature of approximately 22 °C, and a relative humidity of approximately 50%. These studies were performed according to institutional guidelines and approved by the Ethics Committee of the Animal and Plant Quarantine Agency (accreditation number: IACUC-2021-584).

We used MDA(+) (FMD-seropositive) and MDA(−) (FMD-seronegative) wild pigs in the experiments to evaluate the immunogenicity of the antigens isolated and purified from the immune-enhancing FMD vaccine strains, O PA2-C3d and A22-C3d, and assessed their ability to induce an adaptive immune response and overcome MDA interference. The compositions of the vaccines were as follows: a total of 1 mL of vaccine was considered 1 dose, and contained O PA2 + A22 antigens (15 μg + 15 μg; PC group, n = 6/group) or O PA2-C3d + A22-C3d antigens (15 μg + 15 μg; experimental group, n = 6/group), ISA 206 (50% w/w), 10% Al(OH)_3_, and 150 μg Quil-A. Animals in the NC group received the same volume of PBS via the same route. During the experiment, 1 mL of vaccine was administered I.M. twice at 28-day intervals (0 and 28 dpv). Blood samples were collected from the vaccinated pigs at 0, 7, 14, 28, 42, 56, 70, and 84 dpv for use in serological assays such as ELISAs (SP O and SP A), VN titer confirmation and isotype specific antibody immunoassay.

To detect SP antibodies in the sera, we used the PrioCheck^TM^ FMDV type O or FMDV type A (catalog no. 7610420 and 7610850 for FMDV type O and FMDV type A, respectively; Prionics AG, Switzerland) kits and the VDPro^®^ FMDV type O or FMDV type A (catalog no. EM-FMD-05 and EM-FMD-03 for FMDV type O and FMDV type A, respectively; Median Diagnostics, Gangwon, Korea) kits. Absorbance in the ELISA plate was converted to a PI value. When the PI value was ≥50% for the PrioCheck^TM^ FMDV kit or ≥40% for the VDPro^®^ FMDV kit, the animals were considered antibody positive.

A virus neutralization test (VNT) was performed according to the OIE manual^68^. The sera were heat-inactivated at 56 °C for 30 min in a water bath. Cell density was adjusted to form a 70% monolayer, and 2X serial dilutions of sera samples (1:8–1:1024) were prepared. The diluted sera samples were then incubated with a 100-tissue culture infectious dose (TCID)_50_/0.5 mL homologous virus for 1 h at 37 °C. After 1 h, an LF-BK (bovine kidney) cell suspension was added to all wells. After 2–3 days, CPE was evaluated to determine the titers, which were calculated as log_10_ of the reciprocal antibody dilution required to neutralize 100 TCID_50_ of the virus^69,70^. FMDV O/PA2 and FMDV A22/IRAQ were used for the VNT.

To detect isotype specific antibody, ELISA for porcine IgG, IgA, and IgM (catalog no. E101-104, E101-102 and E101-117 for IgG, IgA and IgM, respectively; Bethyl Laboratories. Inc., Montgomery, Texas, USA) were performed on sera according to the manufacturer’s instructions. Briefly, one hundred microliters per well of serially diluted sera and standards were added to the appropriate wells, and the plates were incubated at RT for 1 h. After another washing and drying step, 100 μL/well of the 1X biotinylated detection antibodies were added to all wells, and the plates were incubated at RT for 1 h. The wells were washed and patted dry, 100 μL/well of 1X streptavidin-horseradish peroxidase conjugate was added, and the plates were incubated at RT for 30 min. Subsequently, the plates were washed again and dried. The peroxidase was developed with 100 μL/well of 1X TMB solution for 30 min at RT, and the reaction was stopped with 100 μL 2 N H_2_PO_4_. Absorbance was measured within 30 min using a Hidex 300SL spectrophotometer (Hidex, Turku, Finland) set at 450 nm^17,67^.

To evaluate the O PA2-C3d and A22-C3d mediated cellular immune response and related gene expression, porcine PBMCs were isolated from the whole blood of vaccinated pigs (n = 5/group) at the time points described in Fig. 4a according to the method described by Lee et al. and Jo et al.^17,67^. PBMC isolation was performed as described in the *PBMCs isolation* of the method section. All cells were freshly isolated before use, and no cryopreserved cells were used in any experiment.

### RNA isolation, cDNA synthesis, and quantitative real-time PCR

Total RNA was extracted from the purified porcine PBMCs using TRIzol reagent (Invitrogen) and RNeasy Mini Kits (QIAGEN, Valencia, CA, USA). The cDNA was prepared by reverse transcription using a GoScript Reverse Transcription System (Promega, Madison, WI, USA) according to the manufacturer’s instructions. The synthesized cDNAs were amplified using quantitative-real-time PCR (qRT-PCR) on a Bio-Rad iCycler using the iQ SYBR Green Supermix (Bio-Rad)^17,67^. Gene expression levels were normalized to hprt levels and presented as a relative ratio compared to the control values. The primers used in this study are listed in Table S1.

### Statistical analysis

All quantitative data were expressed as the mean ± standard error (SEM) unless otherwise stated. Between-group statistical differences was assessed using two-way ANOVA followed by Tukey’s post hoc test or one-way ANOVA followed by Tukey’s post hoc test. Statistical significance was denoted as follows: ^∗^*p* < 0.05; ^∗∗^*p* < 0.01; ^∗∗∗^*p* < 0.001; and ^****^p < 0.0001. Parametric tests were used to compare different groups. Survival curves were built using the Kaplan-Meier method, and differences were analyzed using the log-rank sum test. The GraphPad Prism 9.1.2 (GraphPad, San Diego, CA, USA) and IBM SPSS (IBM Corp., Armonk, NY, USA) software were used for all statistical analyses.

### Reporting summary

Further information on the experimental design is available in the Nature Research Reporting Summary linked to this article.

## Supplementary information

Supplementary materials

REPORTING SUMMARY

## Data Availability

All data that support the findings of this study are available from the corresponding author upon reasonable request.
