# Supplementary material for: The C3d-fused foot-and-mouth disease vaccine platform overcomes maternally-derived antibody interference by inducing a potent adaptive immunity
Source: NPJ Vaccines. 2022 Jun 28;7:70. doi: 10.1038/s41541-022-00496-8 (PMC9240001; doi:10.1038/s41541-022-00496-8)
Supplement: Supplementary file 1 — Supplementary materials [file 41541_2022_496_MOESM1_ESM.pdf]

Supplementary Table legends

Supplementary Table 1. List of primer sequences for qRT-PCR.

Supplementary Figure legends

Supplementary Figure 1. Detection of the inactivated FMDV (O PA2, O PA2-C3d, A22, and A22-C3d) antigens in serially dilutions using a rapid test kit for type O or type A.

Structural proteins (SPs) of the purified antigen, expressed by cells infected with the immunostimulatory recombinant FMDV O PA2, O PA2-C3d, A22, and A22-C3d antigens, and confirmed by rapid antigen kits (PBM kits). The images show band formation for the SPs and no band formation for the non-structural proteins (NSPs) of FMDV. (**a**-**d**) O PA2 (**a**); O PA2-C3d (**b**); A22 (**c**); A22-C3d (**d**).

Supplementary Figure 2. Electron microscopic examination of the inactivated antigen of the immunopotent FMD vaccine strain.

The virus particle (146S), as characterized by transmission electron microscope (TEM) imaging. (**a**-**d**) O PA2 (**a**); O PA2-C3d (**b**); A22 (**c**); A22-C3d (**d**).

Supplementary Figure 3. VP1 sequences of the O PA2-C3d and A22-C3d.

VP1 sequence of the O PA2-C3d and A22-C3d adopted on 1st (Sus. 1) and 4th (Sus. 4) suspension cell (BHK) passages were aligned using SnapGene. (**a**-**d**) O PA-C3d nucleotide sequence (**a**); A22-C3d nucleotide sequence (**b**); O PA2-C3d amino acid sequence (**c**); A22-C3d amino acid sequence (**d**).

Supplementary Figure 4. Cellular immune response induced by treatment of O PA2-C3d and A22-C3d on murine peritoneal exudate cells (PECs) and porcine peripheral blood mononuclear cells (PBMCs).

O PA2-C3d and A22-C3d antigen-specific IFNγ secretion were assayed to evaluate cellular immune responses induced by immune-enhancing FMDV antigen using ELISpot assay on

murine PECs and porcine PBMCs. The data were presented as spot forming unit (SFU) of the mean±SEM of triplicate measurements (n=3/group). (**a**, **b**) IFNγ secreting cell spot and SFU in murine PECs (**a**); IFNγ secreting cell spot and SFU in porcine PBMCs (**b**). Statistical analyses were performed using one-way ANOVA followed by Tukey's test. $^{**}p<0.01$; and $^{****}p<0.001$.

Supplementary Table 1

Target	Forward/Reverse	Sequence (5'- 3')	Length(mer)
IFNα	IFNα F	CATCTGCTCTCTGGGCTGTG	20
	IFNα R	TGAGGGGATCCAAAGTCCCT	20
IFNβ	IFNβ F	TGCAACCACCACAATTCCAGA	21
	IFNβ R	GGTTTCATTCCAGCCAGTGC	20
IFNγ	IFNγ F	GCCATTCAAAGGAGCATGGAT	21
	IFNγ R	CTGATGGCTTTGCGCTGGAT	20
IL-1β	IL-1β F	AGCCAGTCTTCATTGTTCAGGT	22
	IL-1β R	TCATCTCTTTGGGGCCATCAG	21
IL-17A	IL-17A F	CTCGTGAAGGCGGGAATCAT	20
	IL-17A R	GGTGTGCTCCGGTTCAAGAT	20
IL-23p19	IL-23p19 F	CCATATCCAGTGCGGGGATG	20
	IL-23p19 R	AGGCCTTGGTGGATCCTTTG	20
IL-23R	IL-23R F	TCCCTCATTGCAAAGCACAA	20
	IL-23R R	GCATCTCCTCTTGCAAGCAAAT	22
IL-2	IL-2 F	AAGCTCTGGAGGGAGTGCTA	20
	IL-2 R	CAACAGCAGTTACTGTCTCATCA	23
IL-10	IL-10 F	CGGCCCAGTGAAGAGTTTCT	20
	IL-10 R	TGCCTTCGGCATTACGTCTT	20
TGFβ	TGFβ F	GGCTGTCCTTTGATGTCACC	20
	TGFβ R	GGCCAGAATTGAACCCGT	18
IL-4	IL-4 F	CTCACCTCCCAACTGATCCC	20
	IL-4 R	TGTGTCCGTGGACGAAGTTG	20
IL-6	IL-6 F	CTGCAGTCACAGAACGAGTG	20
	IL-6 R	CGGCATCAATCTCAGGTGCC	20

Supplementary Table 1 (continued)

Target	Forward/Reverse	Sequence (5'- 3')	Length(mer)
CD40	CD40 F	GTCATCAGCACAAATACTGC	20
	CD40 R	CACAAGTGGTGTCTGTTTTC	20
CD80	CD80 F	TCAGGCATCGTTCAGGTGAC	20
	CD80 R	TGACAGCCAGCACCATTTCA	20
CD86	CD86 F	TGGGACTGAGTAACATTCTCTTTGT	25
	CD86 R	CCAGCTCATCCAGGCTTAGG	20
MHC Class I	MHC Class I F	TGAGCTATTTCTACACCGCCG	21
	MHC Class I R	TCGTCCACGTAGCCGACTT	19
MHC Class II	MHC Class II F	CTCCAGTGATGCTGGGTCAG	20
	MHC Class II R	TGACAGAGTGCCCGTTCTTC	20
CD21	CD21 F	TGCCATGCCTACAAAGCTGA	20
	CD21 R	GTAGTAACCAGGGCGGCATT	20
CD28	CD28 F	TCAAAGGAGTTCCGGGCATC	20
	CD28 R	CTGAAGCAGGCGGGAGTAAT	20
ICOS	ICOS F	GGATGTGCAGCCTTTGTTGT	20
	ICOS R	CAGAGCGTACCAAATTGCGG	20
CTLA-4	CTLA-4 F	GAGTATGGGTCTGCAGGCAA	20
	CTLA-4 R	ATATGTCGCGGCACAGACTT	20
AHNAK	AHNAK F	CACCATCACCGTGACTCGAA	20
	AHNAK R	AGTTCGTGCCGTGGAATCTT	20
HPRT	HPRT F	CCCAGCGTCGTGATTAGTGA	20
	HPRT R	GCCGTTCAGTCCTGTCCATA	20

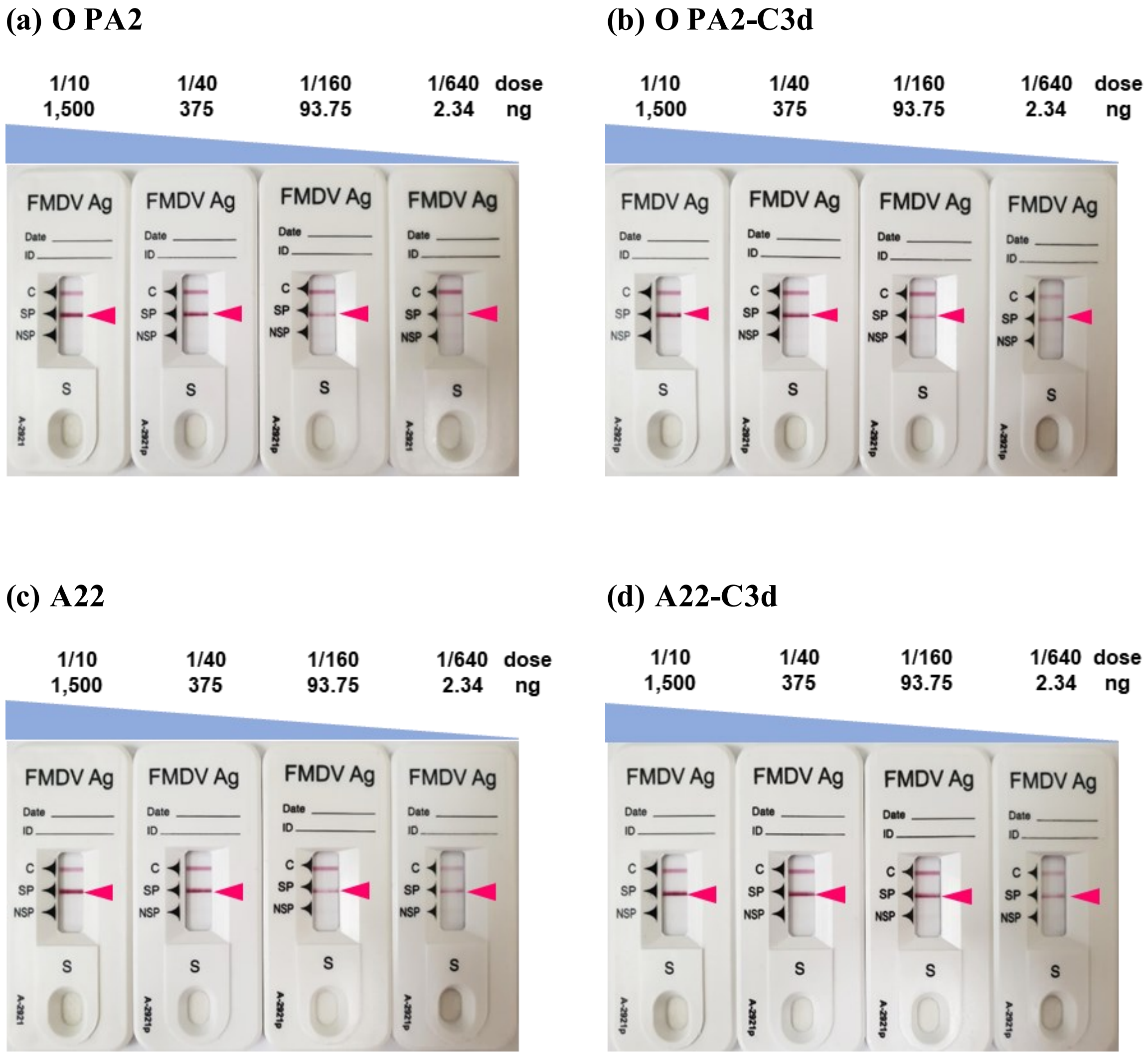

Supplementary Figure 1

(a) O PA2

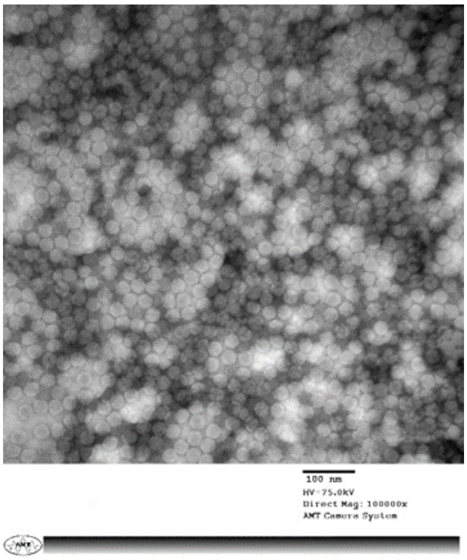

(b) O PA2-C3d

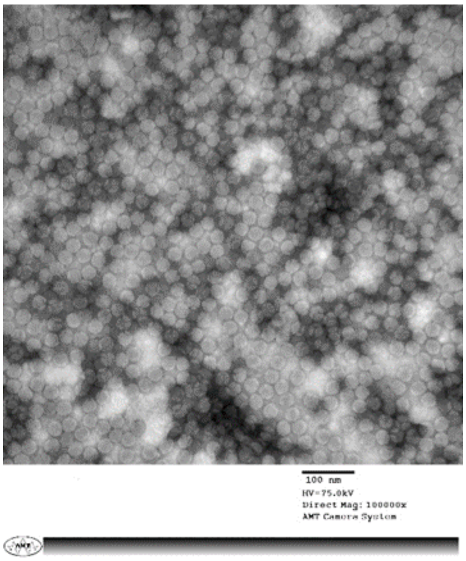

(c) A22

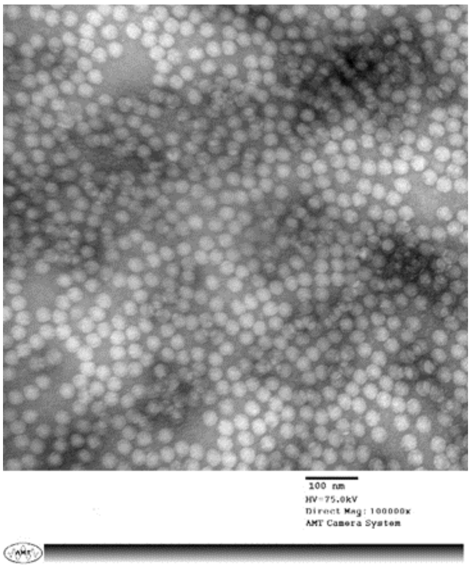

(d) A22-C3d

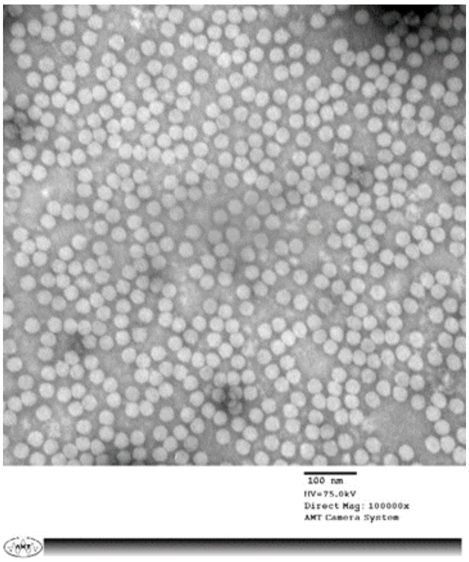

Supplementary Figure 2

(a) O PA2-C3d nucleotide sequence

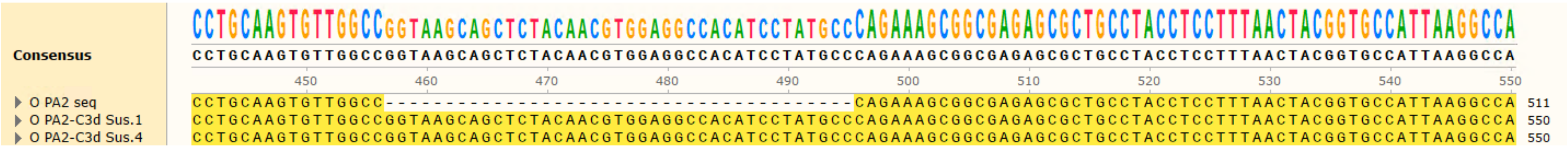

(b) A22-C3d nucleotide sequence

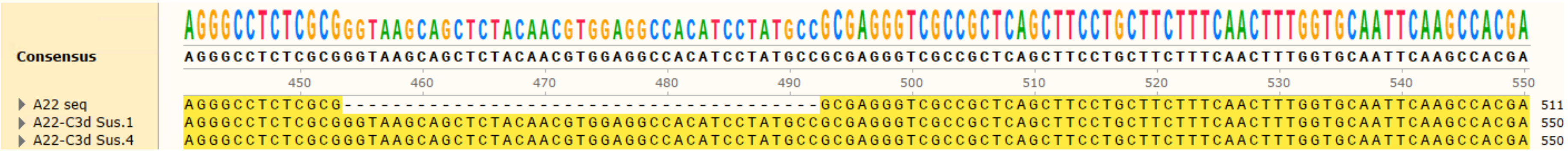

(c) O PA2-C3d amino acid sequence

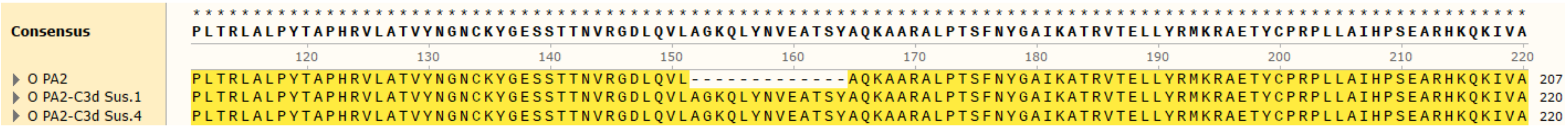

(d) A22-C3d amino acid sequence

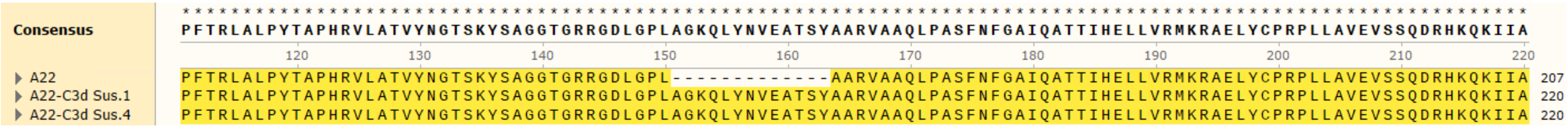

Supplementary Figure 3

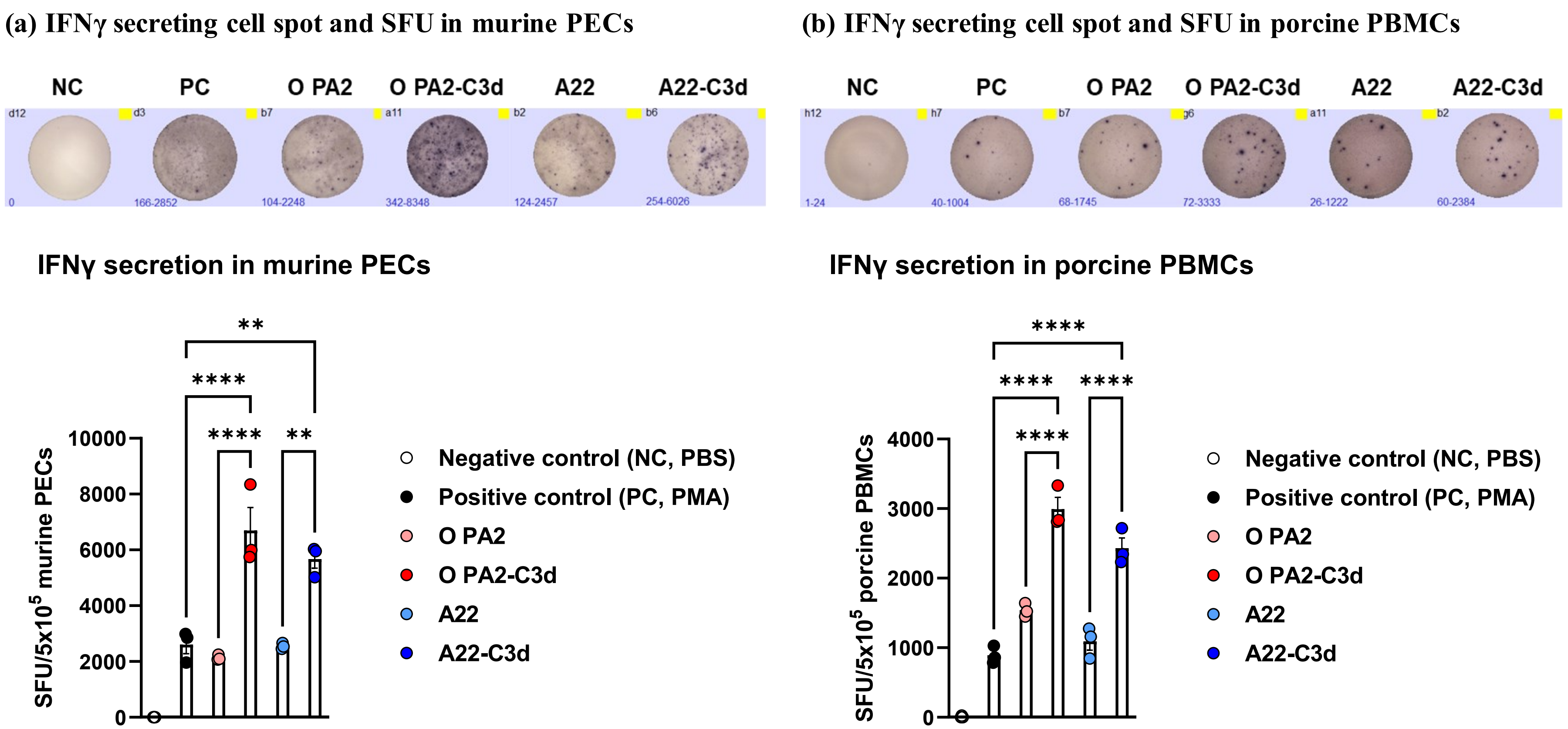

Supplementary Figure 4
